# The posterior cerebellum supports the explicit sequence learning linked to trait attribution

**DOI:** 10.3758/s13415-020-00803-7

**Published:** 2020-06-03

**Authors:** Min Pu, Elien Heleven, Jeroen Delplanque, Noémie Gibert, Qianying Ma, Giulia Funghi, Frank Van Overwalle

**Affiliations:** 1 Faculty of Psychology and Center for Neuroscience, Vrije Universiteit Brussel, Ixelles, Belgium; 2 Faculty of Pharmacy, University of Montpellier, Montpellier, France; 3 Sapienza University of Rome, Roma, Italy

**Keywords:** Cerebellum, Explicit sequence learning, Trait attribution, Metacognition

## Abstract

Recent research has indicated that the cerebellum is responsible for social judgments, such as making trait attributions. The present study investigated the function of the posterior cerebellum in supporting sequence learning linked to trait inferences about persons. We conducted a memory paradigm that required participants to learn a given temporal order of six behavioral sentences that all implied the same personality trait of the protagonist. We then asked participants to infer the trait of the person and to recall the correct order of the sentences and to rate their confidence in their trait judgments and retrieval accuracy. Two control conditions were created: a nonsocial comparison control, involving six nonsocial sentences implying a feature of an object, and a nonsocial nonsequential reading baseline condition. While learning the specific sequence of the sentences, the posterior cerebellum (Crus 2) was more activated for social trait-related sequencing than nonsocial object-related sequencing. Also, given a longer duration to learn the sequences, the precuneus and posterior cingulate cortex were more activated when participants attempted to retrieve the sequences linked to social traits. In addition, confidence in retrieving the correct order of the social sequences modulated the posterior cerebellum (Crus 1) given a longer duration to learn. Our findings highlight the important function of the posterior cerebellum in supporting an active process of sequencing trait-implying actions.

## Introduction

Mentalizing or "mind-reading" refers to the process of inferring the mental states of other people, such as their intentions, beliefs, and traits. To successfully interact with our social environment, people have to predict the mental states and traits of other people. Personality traits are especially helpful for predicting how people will behave (Hassabis et al., 2014). For example, persons characterized as reliable and friendly suggest that we can trust them, whereas aggressive and self-centered persons suggest that we should avoid and distrust them. Although past research on the neural underpinnings of social mentalizing was mainly focused on the cerebral cortex (Schurz & Perner, 2015; Van Overwalle, 2009; Van Overwalle & Baetens, 2009), recent evidence points to the critical role of the cerebellum (Van Overwalle, Baetens, Mariën, & Vandekerckhove, 2014, 2015a; Van Overwalle, D’aes, & Mariën, 2015b).

In a large-scale meta-analysis of more than 350 functional magnetic resonance imaging (fMRI) studies, Van Overwalle et al. (2014) found evidence for the activation of the cerebellum during social mentalizing, including inferences on the intentions, beliefs, and traits of other people. Moreover, strong neural connectivity between the cerebellum and cerebrum during social understanding was revealed in a meta-analytic connectivity study (Van Overwalle, D’aes, et al., 2015b) and across five fMRI studies involving traits and counterfactuals (Van Overwalle and Mariën, 2016; Van Overwalle, Van de Steen, & Mariën, 2019b). More generally, using resting-state connectivity for a total sample of 1,000 participants, researchers found a distinct mentalizing/default network in the cerebellum that was connected to a similar network in the cerebrum (Buckner, Krienen, Castellanos, Diaz, & Yeo, 2011). Together, these studies indicated that the cerebellum is recruited during the social mentalizing, and connected to the mentalizing/default network in the cerebrum (Van Overwalle & Baetens, 2009).

### Cerebellum and sequencing

Despite the rising support for the important role of the cerebellum in social cognition, its underlying neural function remains unexplored. One interesting suggestion is that the cerebellum is critical in acquiring and predicting sequences in motor processing (Molinari et al., 1997), a function that also has been exploited for cognitive reasoning in recent human evolution (Caligiore, Arbib, Miall, & Baldassarre, 2019; Doyon et al., 1997; Ito, 2013; Leggio & Molinari, 2015; Pickering & Clark, 2014; Timmann et al., 2004). In particular, the “sequencing” hypothesis proposed by Leggio and Molinari (2015) states that the cerebellum learns and memorizes patterns of temporally structured events and creates internal models that can be used to make predictions on the sensory and proprioceptive effects of these motor sequences. Studies from cerebellar patients have supported this hypothesis. For example, patients with cerebellum damage were impaired in detecting and repeating motor sequences (Molinari et al., 1997), detecting sequence violations (Restuccia, Della Marca, Valeriani, Leggio, 2007), and in reconstructing the correct sequence of events (Leggio et al., 2008) and behaviors involving complex human actions (Cattaneo et al., 2012). Moreover, in a recent neuroimaging study, participants were asked to complete a Sternberg task that included repeating and novel letter sequences. The results indicated that activations in cerebellar regions contribute to verbal working memory by generating predictions of letter sequences, which supports the hypothesis of sequence learning, detection, and prediction (Peterburs, Blevins, Sheu, & Desmond, 2019).

It therefore seems intuitively plausible that the cerebellum also plays a critical role in the sequencing of social actions. We surmise that for social cognition, the cerebellum builds internal models of social action sequences together with predictions on emotional and behavioral reactions of others and the self. For instance, it makes a great difference in our impressions and reactions whether a person’s aggressive action came first (in an impulse) or after a forceful attack by someone else (in defense). However, in prior research testing the sequence hypothesis, participants were not instructed to infer the mental states of other people, and it thus remains unclear whether sequence identification and generation engage the cerebellum also during social mentalizing. This study investigated whether the cerebellum is involved in learning sequences of actions that inform about the traits of people.

### Cerebellum and mentalizing

Taking into account the increasing evidence that the human cerebellum is recruited during action sequencing, researchers explored the possibility of sequence detection in social mentalizing. A recent pilot study demonstrated that cerebellar patients showed deficits in reconstructing the correct sequence of randomly ordered cartoon-like pictures that require mentalizing about another person’s belief (Van Overwalle, Coninck, Heleven, & Perrotta, 2019b). An fMRI study explored the role of the cerebellum in the same task and documented stronger activation in the posterior cerebellum during identification of action sequences that involved understanding another person’s belief in comparison with non-social mechanical sequences (Heleven, van Dun, & Van Overwalle, 2019). This highlights the critical role of the posterior cerebellum during mentalizing sequences. Importantly for the present study, in the previously mentioned study on cerebellar patients (Van Overwalle et al., 2019a, b; Van Overwalle, Van de Steen, van Dun, & Heleven, 2020a), it was found that they performed marginally worse than healthy controls when making trait judgments based on behavioral descriptions.

Trait attribution rests on the ability to integrate multiple behaviors in a single judgment about the person. The integration of a series of behaviors to arrive at a trait attribution may require the role of the cerebellum. Specifically, to make trait judgments adequately, people have to integrate the orders of actions into a meaningful impression and update the information that existed in previous patterns. As we illustrated earlier, acting first on an impulse (e.g., give a spontaneous slap) or in response to another person’s actions (e.g., in defense) might change trait inferences dramatically. A recent study demonstrated the role of the cerebellum when actions repeated the same trait implication or not (Van Overwalle, Heleven, Ma, & Mariën, 2017) Accordingly, the cerebellum may allow people to learn and anticipate action sequences during social interaction, and automatically detect inconsistencies. The present study investigated the specific sequencing role of the cerebellum in high-level trait mentalizing.

### Present Research

Previous research on the role of the posterior cerebellum during mentalizing (Heleven et al., 2019; Van Overwalle et al., 2019a, b, 2020a) was limited in that it involved single events in which the correct sequence was an inherent part of the social action. Thus, identification of the sequences was mandated by the action elements themselves (Leggio et al., 2008; Van Overwalle et al., 2019a, b, 2020a). It thus remains unclear whether the cerebellum also is involved when learning specific sequences of social events on a larger time scale, that is, when a series of actions by a person involves distinct events performed in different social contexts. The ability to monitor and remember the correct order of social actions that are relatively unrelated is crucial to predict others’ actions, and mixing up the order of such an event is considered socially inept.

In the present study, we investigated this question of larger-scale action series. We explored whether the cerebellum is recruited while learning the sequence of a series of events that are arbitrarily ordered and without an inherent logical order. However, instead of being totally unconnected, the actions were related in that they all implied the same personality trait of the agent, and participants were instructed to infer this trait. For instance, giving a compliment, buying a present, listening to someone, and so on, are all distinct actions, but they are related by a similarly implied trait, namely kindness. Instructing the participants to infer a common trait from a sequence of actions is a novel, but critical element of our design, because this ensures that learning action sequences triggers a social mentalizing process, not merely a cognitive nonsocial process. To summarize, in the present experiment, participants were requested to memorize a given sequence of action sentences in order to recruit the cerebellum. Moreover, this was done in a context of a trait inference process to recruit the posterior part of the cerebellum responsible for social mentalizing. However, because all actions implied the same trait, any order of the actions was of no consequence for the trait attribution, so that the sequencing and attribution manipulations were in principle unconfounded.

In addition, we investigated how metacognitive experiences develop while retrieving action sequences. Successful social interaction and adaptive behavior depend on accurate monitoring and controlling our cognitive processes, which is known as “metacognition.” Neuroimaging studies indicate that domain-general neural substrates (i.e., frontolateral and frontomedial cortex) contribute to metacognitive processes across the perception and memory domain (Baird et al., 2013; Fleming et al., 2010; Maniscalco, McCurdy, Odegaard, & Lau, 2017; see meta-analysis by Vaccaro & Fleming, 2018). However, there also is preferential engagement of brain areas related to the processes underlying specific tasks (Vaccaro & Fleming, 2018). This suggests that frontal areas represent a central hub integrating signals originating from local activations in areas recruited for each specific task. We investigated the neural substrates of metacognitive confidence on sequence retrieval during mentalizing.

To summarize, the current study investigated the role of the cerebellum in learning and recognizing sequences during trait mentalizing. We applied a memory paradigm that required participants to learn a given temporal order of distinct actions that all implied the same personality trait. Participants were instructed to infer the trait implied by the behavior. In an immediate memory test, we then asked participants to recall the correct order of events. To investigate how they metacognitively monitor and evaluate their performance, we also asked participants how confident they were when retrieving the correct action sequences and inferring a trait. To investigate the unique role of social mentalizing, we created a matched nonsocial comparison condition in which sentences implied a feature of an object rather than a trait of a person. In addition, we added a baseline condition without any sequencing, requiring participants to simply read and understand nonsocial sentences.

We hypothesized that acquiring and learning the order of several events implying personality traits requires a strong involvement of the posterior cerebellum compared with the nonsequence reading baseline condition and nonsocial sequencing condition. We further hypothesized that metacognitive confidence in retrieving the correct order of actions will recruit lateral or medial frontal cortical areas, potentially with the aid of local activation in posterior cerebellar areas involved in sequencing. We did not have specific hypotheses with respect to the retrieval of the order of (non)social events after learning. The reason is that cerebellar sequence theories assume that the main function of the cerebellum is to reduce errors and therefore predict strong cerebellar activation during initial training of sequences when the likelihood of errors is high but not on retrieval when learning is close to completion and the likelihood of errors is low (Caligiore et al., 2019; Doyon et al., 1997; Ito, 2013; Leggio & Molinari, 2015; Pickering & Clark, 2014; Timmann et al., 2004).

## Method

### Participants

Twenty-seven healthy, right-handed, native Dutch-speaking volunteers were recruited to take part in the fMRI study. Two participants who failed to complete the whole experiment were excluded. Thus, 25 participants (19 females; age mean ± SD, 23 ± 3 years) were included in the analysis. All participants had normal or corrected-to-normal vision and reported no neurological or psychiatric disorders. Informed consent was obtained with the approval of the Medical Ethics Committee at the Hospital of University of Ghent, where the study was conducted. Participants were paid 20 euros in exchange for their participation and transportation costs.

### Stimulus materials

We presented sets of six sentences each. The social trait-implying sentence sets described a fictitious protagonist engaging in a series of behaviors from which a strong trait (e.g., honest, unfriendly) could be inferred. The nonsocial sentence sets described an object involved in a series of events that implied a strong feature, for example, “the necklace has diamond decorations” implies a feature “valuable” of the “necklace.” The sentences and traits/features were copied from earlier trait-implying research (Ma et al., 2014; Van der Cruyssen, Heleven, Ma, Vandekerckhove, & Van Overwalle, 2015) or constructed anew by the authors. We also created novel distractor trait/features for all these sentences that were less applicable than the original trait/feature, but with the same valence. Next, this material was pilot tested by asking participants (n = 16-30 for different parts of the material) to rate how applicable the trait/feature and distractors were with respect to all events described in each sentence set, using a 7-point scale (1 = *not applicable at all*; 4 = *neutral*; and 7 = *very applicable*). Correct traits and features were selected when the applicability rating was >6, whereas distractors were selected when the applicability rating was at least 2 scale-points lower than the correct trait/feature. All selected sentences contained 5 to 10 words, with most sentences having 7 words.

### Procedure

For the experimental conditions, we conducted a 2 Domain (social vs. nonsocial) by 2 Duration (20 seconds vs. 40 seconds) design. We applied a memory paradigm from a previous study investigating metacognition in memory (McCurdy et al., 2013). Participants were instructed to learn the given temporal order of a set of six sentences involving a single person or object and had to infer from these six sentences a common trait of the person or common feature of the object. There were 16 social trait sentences sets and 16 nonsocial feature sentence sets in total, and their order had to be learned in 20 seconds for half of them and in 40 seconds for the other half (randomly determined). The two different durations (20 and 40 seconds) were created to induce different levels of difficulty so that this would generate varying levels of retrieval performance and metacognitive confidence (see also McCurdy et al., 2013). The order in which the social and non-social sentence sets were presented, as well as the order in which the six sentences of each set were presented, were randomly determined for every participant.

**Fig. 1:**
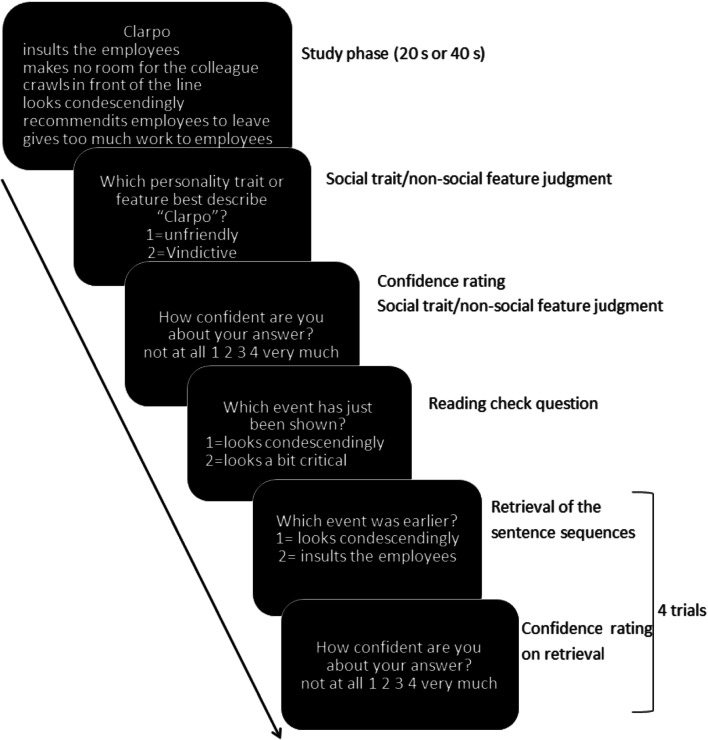
Experimental procedure. Participants were instructed to learn the given temporal order of a set of six sentences involving a single person or object and had to infer from these six sentences a common trait of the person or feature of the object. All question were preceded by a blank screen with fixation cross in the center which was jittered randomly between 0-2 s, and ratings had to be answered within 5 s

The experimental task is illustrated in Fig. 1. For each sentence set, the same procedure was followed (before the actual experiment, participants performed several practice trials on this task).

At the beginning, participants were instructed to learn and memorize the correct temporal order of a set of sentences shown on screen *(study phase).* For each sentence set, the name of the main person or object appeared on the top of the screen and six sentences were shown on screen one-by-one for 1.1 seconds (timing determined by pilot testing) and were then presented together on the screen for a total duration of 20 seconds or 40 seconds (including the initial one-by-one reading). A red notice appeared on the top to inform participants that only 10 s were left to learn the sentence orders. This timing was determined by pilot testing, in which 21 participants underwent the same study outside the scanner, and obtained an overall retrieval accuracy of 77%.

After the study phase, to ensure that participants made trait inferences during the study phase, they were asked “Which personality trait does describe the person best?” or “Which feature does describe the object best?” Two possible options were shown on the screen in a random order, involving the correct trait/feature and a distractor with the same valence. Immediately afterwards, participants were asked to rate how confident they were about their trait/feature judgment using a 4-point scale (1 = *not at all*; 4 = *very much*). Next, participants received one factual question as manipulation check to verify whether they had read and understood the sentences. They read one original sentence of the set, as well as another sentence with was very similar in content and phrasing, but with at least one different word (often a semantically related word). Participants were instructed to respond which of the two sentences was shown before (1 = *the first sentence*; 2 = *the second sentence*).

Finally, participants had to retrieve the correct order of the sentences in a memory task consisting of four trials *(retrieval phase)*. On each trial, they read two sentences selected randomly from the sentences set and shown a random order, and they had to indicate the order in which the two sentences were shown earlier during the study period (1 = *the first sentence*; 2 = *the second sentence*). Immediately after each trial, they were asked to rate how confident they were about their retrieval judgment (*metacognitive* phase), using a 4-point scale (1 = *not at all*; 4 = *very much*).

All question and ratings had to be answered within 5 seconds. All questions were preceded by a blank screen with a fixation cross in the center, which were jittered randomly between 0 ms to 2,000 ms (mean = 1,000 ms). All responses were given on a response box with the (nondominant) left hand. When a trait judgment or the related confidence rating was missing, this trial was excluded from the analysis. Likewise, when a retrieval judgment was missing or the related confidence rating was missing, this trial was excluded from the analysis.

To provide a general baseline for the scanner, we included a reading baseline control condition at the start of the experiment, during which participants simply read sets of nonsocial sentences without remembering their order. These sets had to be read in 20 or 40 seconds (randomly determined), although participants were allowed to end the trial earlier, because simply reading the sentences typically took less time. All other aspects of the procedure were identical to the experimental conditions, including feature ratings and related confidence ratings, as well as manipulation checks, except that no retrieval task and related confidence rating was provided. There were eight sets of reading baseline sentences identical to the number of sets in each of the four experimental conditions.

To summarize, the design involved three main conditions (social sequencing, nonsocial sequencing, and nonsocial nonsequencing conditions). When considering the main conditions in this order, all critical comparisons of the main social sequencing condition with appropriate control conditions are possible. First, to test whether the sequencing role of the cerebellum is specific to social mentalizing, a nonsocial sequencing control condition was included. Second, to test whether sequencing was critical for cerebellar activation, we included the nonsocial nonsequencing baseline control condition.

### Questionnaire

We examined whether metacognitive sensitivity on retrieving the sequences was correlated with individual differences in alexithymia, which reflects a lack of self-awareness on experienced emotionality. After participants left the scanner, they completed the Dutch version of the 20-item Toronto Alexithymia Scale (TAS-20) (Bagby, Taylor, & Parker, 1994; Dutch version: Bermond, Vorst, & Oort, 1998).

### Imaging procedure and preprocessing

Images were collected with a Siemens Magnetom Prisma fit scanner system (Siemens Medical Systems, Erlangen, Germany) using a 64-channel radiofrequency head coil. Stimuli were projected onto a screen at the end of the magnet bore that participants viewed by way of a mirror mounted on the head coil. Stimulus presentation was controlled by E-Prime 2.0 (www.pstnet.com/eprime; Psychology Software Tools) running under Windows XP. Participants were placed head first and supine in the scanner bore and were instructed not to move their heads to avoid motion artifacts. Foam cushions were placed within the head coil to minimize head movements. First, a high-resolution anatomical images were acquired using a T1-weighted 3D MPRAGE sequence [TR = 2250 ms, TE = 4.18 ms, TI = 900 ms, FOV = 256 mm, flip angle = 9°, voxel size = 1 × 1 × 1 mm]. Second, a fieldmap was calculated to correct for inhomogeneities in the magnetic field (Cusack & Papadakis, 2002). Third, whole brain functional images were collected in a single run using a T2*-weighted gradient echo sequence, sensitive to BOLD contrast (TR = 1,000 ms, TE = 31.0 ms, FOV = 210 mm, flip angle = 52°, slice thickness = 2.5 mm, distance factor = 0%, voxel size = 2.5- × 2.5- × 2.5-mm, 56 axial slices, acceleration factor GRAPPA = 4).

SPM12 (Wellcome Department of Cognitive Neurology, London, UK) was used to process and analyze the fMRI data. To remove sources of noise and artifact, data were preprocessed. Inhomogeneities in the magnetic field were corrected using the fieldmap (Cusack & Papadakis, 2002). Functional data were corrected for differences in acquisition time between slices for each whole-brain volume, realigned to correct for head movement, and co-registered with each participant’s anatomical data. Then, the functional data were transformed into a standard anatomical space (2-mm isotropic voxels) based on the ICBM152 brain template (Montreal Neurological Institute). Normalized data were then spatially smoothed (6-mm full-width at half-maximum, FWHM) using a Gaussian Kernel. Finally, using the Artifact Detection Tool (ART; http://web.mit.edu/swg/art/art.pdf;http://www.nitrc.org/projects/artifact_detect), the preprocessed data were examined for excessive motion artifacts and for correlations between motion and experimental design, and between global mean signal and experimental design. Outliers were identified in the temporal differences series by assessing between-scan differences (Z-threshold: 3.0 mm, scan to scan movement threshold: 0.5 mm; rotation threshold: 0.02 radians). These outliers were omitted from the analysis by including a single regressor for each outlier. A default high-pass filter was used of 128s and serial correlations were accounted for by the default auto-regressive AR(1) model.

### Statistical analysis of neuroimaging data

The general linear model of SPM12 (Wellcome Department of Cognitive Neurology, London, UK) was used to conduct the analyses of the fMRI data. At the first (single participant) level, the event-related design was modeled with one regressor for each condition (social 20 seconds, social 40 seconds, nonsocial 20 seconds, nonsocial 40 seconds, reading baseline). During the study phase*,* onsets in this model were specified at the presentation of the first sentence, fourth sentence, and all sentences-at-once of the sentence set. After the study phase, onsets in this model were specified at the presentation of each question (trait, trait confidence, retrieval, retrieval confidence). We did not model the responses as a separate regressor. As mentioned earlier, missed trials were not modeled.

Each regression was convolved with a canonical hemodynamic response function of which the duration was set to 0 s for all questions and ratings after the study phase. During the study phase, duration was determined as follows:

During the reading baseline, event duration for reading all sentences in the study phase was set to 4 s. An analysis of the reading times revealed that 4 s was on average the shortest reading time needed to read the baseline control sentences. Sentence sets with reading time shorter than 4 s were excluded from the fMRI analysis, the mean rejection rate of sentence sets is 10% (SD = 13%).

During the experimental conditions, in the study phase event duration was set to 10 s for reading the social and nonsocial sentences and for memorizing their order. We reasoned that during the study phase, encoding the sentence order would primarily take about 10 seconds, while the remaining time would be used for rehearsal; 4 seconds would probably be too short to capture the encoding of the sentence order. Exploratory comparisons between 4 s and 10 s durations indeed revealed stronger effects in the posterior cerebellum under the longer 10 s duration, which was therefore kept for the main analyses.

At the second (group) level, clusters from whole-brain analyses were defined at threshold *p* < 0.001, uncorrected with a minimum cluster extent of 10 voxels, and we restricted the analysis further to clusters with a Family Wise Error (FWE) corrected cluster-wise threshold *p* < 0.05. For all study phases and all questions, we conducted a within-participant ANOVA and defined all possible t-contrasts of interest between Domain (social vs. non-social) and Duration (20 s vs. 40 s). In addition, to ensure that trait inferences ware made, we conducted a repetition suppression analysis where we looked for a decrease in activation of the neural substrates coding for trait information, by conducting a 1^st^ > 4^th^ sentence contrast (for similar analyses, see Heleven & Van Overwalle, 2016; Ma et al., 2014). In line with the logic of these studies, suppression of activation in the mPFC indicates that trait inferences were being made while learning the sequences of actions.

Because whole-brain activity between the social and nonsocial domains was only marginally significant in the cerebellum, we explored these differences further using regions of interest (ROI) with centers based on earlier meta-analyses on social mentalizing and the cerebellum (MNI coordinates ±24 -76 -40; Van Overwalle & Mariën, 2016; Van Overwalle et al., 2019b, 2020a). A sphere of 15-mm radius around the centers was used to perform a small volume correction using the same thresholds as the whole-brain analysis.

We also conducted parametric analyses to investigate whether brain activity covaried with retrieval accuracy or with the confidence ratings. To this aim, retrieval accuracy and behavioral confidence ratings were included in first-level analyses as a parametric modulator on a trial-by-trial basis (Fleming, Huijgen, & Dolan, 2012; Morales, Lau, & Fleming, 2018). Separate regressors were created for each experimental condition. Specifically, we ran two parametric models: 1) during the retrieval phase with accuracy of retrieval (correct or incorrect) as parametric modulator, and 2) during the confidence rating phase with confidence rating as modulator. Single-subject contrast images of the parametric modulator were entered into a second-level, random-effects analysis, which was similar to the one described above.

We also conducted second level regression analyses to investigate whether brain activity covaried with retrieval accuracy or confidence at the individual level. Specifically, to examine whether activation during sequence retrieval was correlated with individual indices of retrieval accuracy, each participant’s mean retrieval accuracy was entered as covariate in a second-level regression analysis in each of the four experimental conditions (Bègue et al., 2019). Likewise, to examine whether activation during confidence judgments on sequence retrieval was correlated with individual indices of metacognitive ability, each participant’s meta-ratio index was entered as covariate in a second-level regression analysis in each of the four experimental conditions (Bègue et al., 2019). This latter analysis revealed activation in the cerebellum Crus 1 in the social 40-s condition. To support and clarify this result, we also extracted the percentage signal change of Crus 1 in this condition using the MarsBar toolbox (http://marsbar.sourceforge.net), using a sphere as region of interest centered at the Crus 1 peak coordinates (Table 4) with a radius of 8 mm.

Because we had no specific hypotheses on metacognitive confidence on trait attributions, for exploratory reasons, we conducted a similar whole-brain analysis as above. Because participants were equally highly confident about the trait and object judgment (showing a ceiling effect on confidence ratings), and the ratings involved only four discrete scale points (1-4) and thus are not continuous; first-level parametric and second-level regression analyses were not conducted.

### Statistical analysis of behavioral data

We analyzed accuracy of the selected trait/feature, accuracy of retrieval memory (quantified as the % correct responses in a sentence set), confidence ratings of retrieval performance, and manipulation check questions. For the analysis of confidence ratings, we removed trials with missed responses.

To estimate metacognitive efficiency, we used the meta-d' calculation (Maniscalco & Lau, 2012) on participants’ confidence rating, using scripts that are publicly available (http://www.columbia.edu/~bsm2105/Type2sdt/). Meta-d' is widely known as the measurement of metacognitive sensitivity (type 2 sensitivity) in a signal detection (SDT) framework and is expressed on the same scale as the type 1 sensitivity metric d' reflecting objective performance to allow direct comparisons (Fleming & Lau, 2014). We also calculated meta-ratio (meta-d'/d'), which tests the efficacy of metacognitive sensitivity by qualifying the degree to which confidence ratings discriminate between correct and incorrect trials unconfounded by first-order performance (Fleming et al., 2012; Fleming & Lau, 2014). In particular, to test metacognition on retrieving the sequences, we estimated meta-ratio (meta-d'/d') for metacognition on retrieving the sequences and trait judgment as well.

## Results

### Behavioral results

For social trait questions, the average accuracy of trait judgment was 94% (SD = 7%), and the mean accuracy of the check question was 84% (SD = 12%). For nonsocial object-feature questions, the average accuracy of feature judgment was 94% (SD = 8%), and the mean accuracy of the check question was 82% (SD = 11%).

The average retrieval performance was 76% (SD = 12%) across the social and non-social conditions. A 2 (Domain: social vs. nonsocial) by 2 (Duration: 20 s vs. 40 s) repeated measures ANOVA on retrieval accuracy revealed a main effect of Duration (F(1,24) = 8.59, *p* = 0.007, *η*^2^_*p*_ = 0.26), with retrieval performance in the 40-s condition (mean ± SE: 78% ± 2.6%) being significantly higher than that in the 20-s condition (mean ± SE: 74% ± 2.8%). The main effect of Domain was not significant, F(1,24) = 0.31, *p* = 0.58, *η*^2^_*p*_ = 0.013, with identical accuracy in both social (mean ± SE: 76% ± 2.8%) and nonsocial (mean ± SE: 75% ± 2.8%) conditions. The interaction between Duration and Domain was not significant (*p* > 0.1). Overall, the participants missed 8% of the retrieval trials in each of the social and nonsocial conditions, and they missed 2% of the confidence ratings in the memory task in each of the social and nonsocial domains (these missed trials were excluded from the fMRI analysis).

We found that the mean meta-d'/d' for the social and nonsocial conditions was 2.38 (SD = 5.23) and 1.08 (SD = 3.12), respectively. A paired *t*-test comparing meta-d'/d' on the social and nonsocial conditions found no significant differences, *t*(24) = 1.07, *p* = 0.29, indicating that metacognition was equally efficient when participants retrieved social and nonsocial sentence sequences. We then conducted a 2 (Domain: social vs. nonsocial) by 2 (Duration: 20 s vs. 40 s) repeated measures ANOVA on meta-ratio, but no significant effects were found. However, given that the standard deviations were larger than the original study by McCurdy et al. (2013), these nonsignificant results should be treated with some caution and require attention in future research. As might be expected, we found a robust negative correlation of alexithymia with metacognitive ability (meta-d') on retrieving the sequences, Spearman *r* = −0.56, *p* = 0.003.

For metacognition on trait judgment, we found that the mean meta-d'/d' for the social and non-social conditions was 0.95 (SD = 0.54) and 1.24 (SD = 0.41), respectively. A paired *t*-test comparing meta-d'/d' on the social and nonsocial conditions found a significant difference with higher meta-ratio in the nonsocial condition than the social condition, *t*(24) = −2.28, *p* = 0.031.

### fMRI results

#### Study phase: Learning sequences of actions

**Fig. 2:**
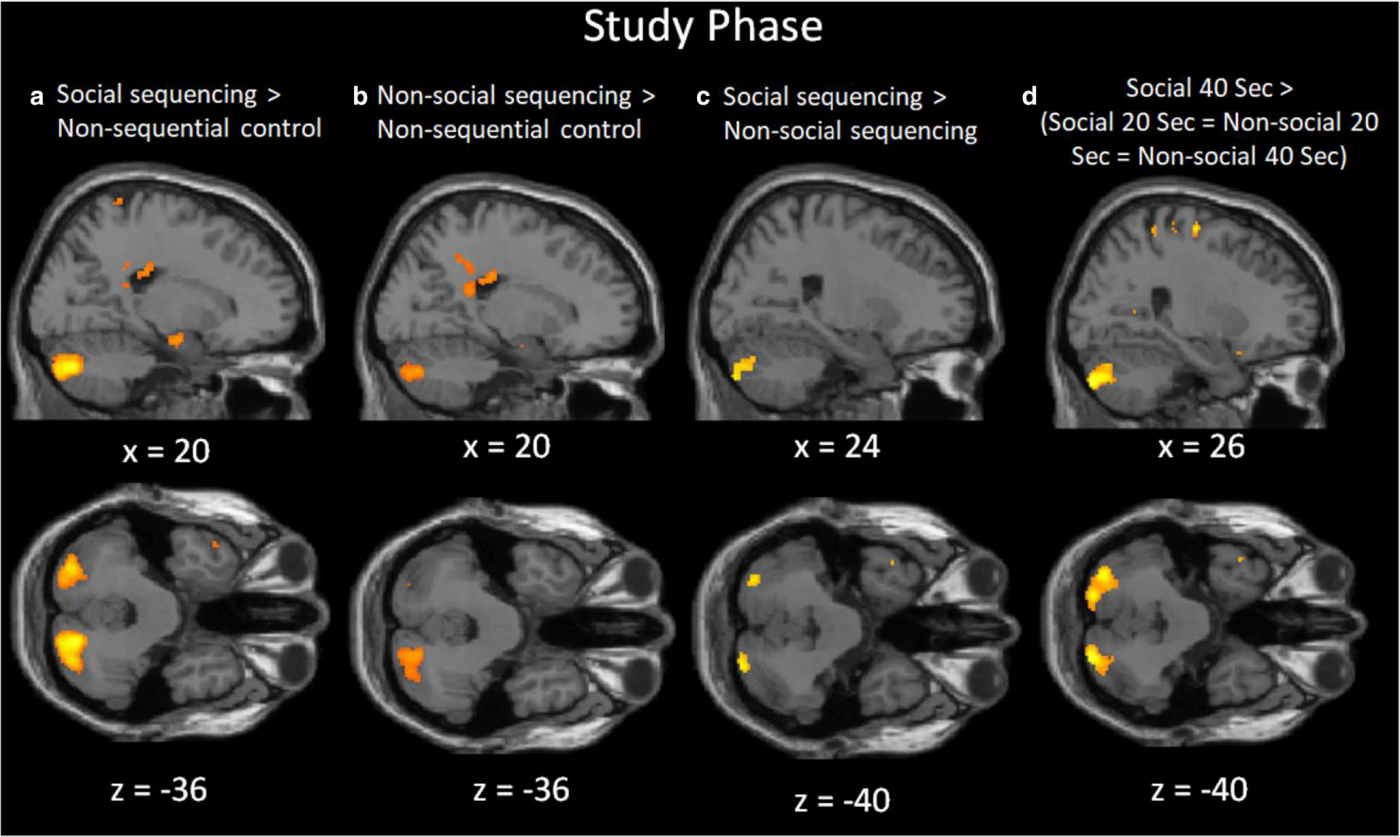
Sagittal and transverse views of the experimental contrasts during the study phase (learning the action sequences) at an uncorrected threshold of *p* < 0.001. Significant clusters (*p* < 0.05, FWE corrected) were found in the posterior cerebellum (Crus II) for the contrasts of (**A**) Social sequencing > Nonsequential control, (**B**) Nonsocial sequencing > Nonsequential control, (**C**) Social sequencing > Nonsocial sequencing, and the interaction (**D**) Social 40 s > Social 20 s = Nonsocial 20 s = Nonsocial 40 s. Note that for (**C**), the contrast was only marginally significant at *p* < 0.08 but was significant after applying a small volume correction centered around the posterior cerebellum (see text)

To investigate whether the cerebellum is involved in learning sequences of social and nonsocial actions, we first compared the experimental sequential conditions (across the social and nonsocial conditions) against the nonsequential reading baseline condition. As expected, this contrast revealed significant posterior cerebellar activation. Additional (sub)cortical activations were found in the middle occipital gyrus, middle temporal gyrus, precuneus and hippocampus (Table 1). Splitting the data by domain, in the social condition, the same contrast revealed significant brain activations in the posterior cerebellum as well as the same (sub)cortical areas (but with posterior cingulate instead of precuneus), and additionally the TPJ and mPFC (Fig. 2A; Table 1). In the nonsocial domain, posterior cerebellar activation was also found, together with activation in the same cortical areas except the hippocampus (Fig. 2B; Table 1).

**Table 1.** Whole-brain analysis of action sequencing during the study phase

Contrasts and Anatomical Label	*MNI coordinate*			*Voxels*	*max t*
	*X*	*y*	*z*		
**Sequencing > Nonsequential Control**					
R Cerebellum (Crus 2)	20	−76	−36	532	5.29***
R Cerebellum (Crus 2)	32	−80	−36		4.58***
L Cerebellum (Crus 2)	−28	−84	−28	320	4.97***
L Cerebellum (Crus 2)	−18	−78	−40		4.24***
L Cerebellum (Crus 2)	−28	−78	−38		4.23***
L Middle Occipital Gyrus	−42	−68	6	1783	8.41***
R Middle Temporal Gyrus	44	−66	4	1315	8.38***
L Precuneus	−6	−50	10	541	5.02***
L Hippocampus	−24	−8	−22	158	5.28*
L Middle Temporal Gyrus	−56	4	−20	162	4.82*
**Social Sequencing > Nonsequential Control**					
R Cerebellum (Crus 2)	20	−76	−36	633	5.94***
R Cerebellum (Crus 2)	32	−78	−34		5.02***
L Cerebellum (Crus 2)	−30	−84	−28	464	5.34***
L Cerebellum (Crus 2)	−30	−78	−36		5.03***
L Cerebellum (Crus 2)	−18	−78	−36		4.39***
L Middle Occipital Gyrus	−42	−68	6	1,967	8.10***
R Middle Temporal Gyrus including TPJ	44	−66	4	1,340	7.83***
L Posterior Cingulate Cortex	0	-52	30	816	6.00***
L Middle Temporal Gyrus	−64	−12	−12	683	5.45***
L Hippocampus	−24	−8	−22	194	5.73*
mPFC	−8	62	26	289	4.48**
**Nonsocial Sequencing > Nonsequential Control**					
R Cerebellum (Crus 2)	20	−76	−36	310	4.32***
R Cerebellum (Crus 2)	30	−80	−32		3.94***
R Middle Temporal Gyrus	46	−68	4	1217	8.47***
L Middle Occipital Gyrus	−42	−68	6	1411	8.22***
L Precuneus	−6	−50	10	225	5.05**
**Social Sequencing > Nonsocial Sequencing**					
R Cerebellum (Crus 2)	24	−86	−40	120	4.07°
L Cerebellum (Crus 2)	−32	−80	−36	123	4.41°
R Angular Gyrus, including TPJ	50	−60	28	221	4.52**
L Angular Gyrus, including TPJ	−40	−56	24	584	5.58***
L Precuneus	−8	−54	38	931	6.16***
R Middle Temporal Gyrus	62	−4	−20	444	5.95***
L Medial Temporal Gyrus	−48	12	−28	1,094	6.08***
mPFC	−2	48	40	342	4.13***
**Nonsocial Sequencing > Social Sequencing**					
---					
**20 s > 40 s (all Sequencing conditions)**					
R Lingual Gyrus	4	−78	−2	10825	10.53***
L Hippocampus	−22	−30	−4	195	6.06*
L Precentral Gyrus	−48	−4	54	6483	6.72***
L Middle Frontal Gyrus	−38	48	12	382	4.57***
**40 s > 20 s (all Sequencing conditions)**					
R Angular Gyrus, including TPJ	54	−64	30	25,8	5.58**
L Angular Gyrus including TPJ	−44	−64	30	340	4.46***
**Social 20 s > Social 40 s**					
R Lingual Gyrus	4	−78	−2	2076	8.12***
R Middle Occipital Gyrus	32	−74	24	585	5.24***
L Fusiform Gyrus	−34	−72	−14	1725	5.86***
L Precentral Gyrus	−48	−4	54	451	5.19***
**Social 40 s > Social 20 s**					
R Angular Gyrus, including TPJ	54	−64	28	223	5.32**
L Angular Gyrus, including TPJ	−44	−64	28	270	4.24**
R Calcarine Gyrus	16	−56	10	194	4.82*
**Nonsocial 20 s > Nonsocial 40 s**					
L Lingual Gyrus	−30	−88	−14	2849	5.59***
R Lingual Gyrus	4	−78	−2	3048	8.35***
R Superior Parietal Lobule	24	−64	50	1326	5.28***
L Precentral Gyrus	−46	−6	56	269	5.45***
**Nonsocial 40 s > Nonsocial 20 s**					
---					
**Spreading Interaction: Social 40 s > (Social 20 s = Nonsocial 20 s = Nonsocial 40 s)**					
R Cerebellum (Crus 2)	26	−84	−40	452	5.24***
R Cerebellum (Crus 2)	34	−78	−36		4.49***
L Cerebellum (Crus 2)	−30	−78	−36	509	5.68***
L Cerebellum (Crus 2)	−20	−86	−36		5.08***
R Angular Gyrus, including TPJ	54	−60	28	585	6.98***
L Angular Gyrus, including TPJ	−42	−58	24	838	6.72***
L Posterior Cingulate Cortex	0	−52	32	1062	6.44***
L Middle Temporal Gyrus	−58	−8	−24	611	6.39***
R Middle Temporal Gyrus	60	−2	−18	320	5.61***
mPFC	−10	38	56	154	5.46*

Coordinates refer to the MNI (Montreal Neurological Institute) stereotaxic space. Whole-brain analysis thresholded at voxel-wise uncorrected *p* < 0.001 with cluster-wise FWE corrected *p* < 0.05, with voxel extent ≥10. Only the highest peaks of each cluster are shown, except for the cerebellum showing all peaks. L = left, R = right

*p < 0.05, **p < 0.01, ***p < 0.001 (peak FWE corrected). °*p* < 0.08, cluster-level FWE corrected and *p* < 0.005 cluster-level FWE corrected using a small volume correction with a sphere with 15-mm radius and centered around a priori MNI coordinates [±24 −76 −40] (Van Overwalle & Mariën, 2016; Van Overwalle et al., 2019b, 2020a, b)

More importantly, we tested the critical difference between social and non-social sequential condition. Although the posterior cerebellar activation for this contrast was only marginally significant in the whole-brain analysis (*p* < 0.08, cluster-wise FWE corrected), the results from the ROI analysis revealed that the bilateral posterior cerebellum was more strongly recruited in the social than non-social sequencing condition (Table 1; Fig. 2C). There were additional whole-brain activations in the precuneus, middle and medial temporal gyrus, angular gyrus including the TPJ, and mPFC (Table 1). These results confirm our prediction that learning sequences linked to trait mentalizing recruits more brain activity in the posterior cerebellum. No significant activity was found in the reverse contrast of nonsocial sequencing > social sequencing.

Moreover, we tested how the duration of sequential learning in the study phase impacts activity in the cerebellum. To do this, we compared the 20 s and 40 s condition across and within the social and nonsocial domain. The results revealed no significant activation in the posterior cerebellum (Crus 2) for this contrast of both directions, nor in each social and on-social domain separately. We also tested the notion that activation in the social 40-s condition was predominantly higher compared with all other conditions (i.e., spreading interaction contrast: social 40 s > [social 20 s = nonsocial 20 s = nonsocial 40 s]). The results indicated increased brain activity in the posterior cerebellum (Crus II) when participants had 40 s to learn the sequences in the social context (Fig. 2D).

#### Trait suppression

To ensure that trait inferences were made during the sequence learning task, we conducted a whole-brain, random-effects analysis contrasting prime (the first sentence) > target (the fourth sentence) trials in the social and nonsocial domains, separately. The whole-brain analysis of the prime > target contrast in the social domain showed significant suppression effects in the ventral part of the mPFC (Table 2). Additional brain regions were found in the middle and superior temporal gyrus, and lingual gyrus. This repetition suppression effect was also observed in the non-social domain, as indicated by the increased vmPFC activation for the contrast of prime > target (Table 2). We also observed activation in the middle and superior temporal gyrus, middle frontal gyrus, middle anterior cingulate and occipital gyrus (Table 2). We also sought for any interaction effects in repetition suppression between social and nonsocial domain, but no significant activation was found.

**Table 2.** Whole-brain analysis of trait suppression

Contrasts and Anatomical Label	*MNI coordinate*			*Voxels*	*max t*
	*x*	*y*	*z*		
**Social Trait Prime > Target**					
R Lingual Gyrus	14	-72	-6	12591	8.58***
R Middle Temporal Gyrus	52	-66	6	2019	6.95***
L Middle Temporal Gyrus	-42	-64	6	456	6.12***
L Superior Temporal Gyrus	-58	-4	4	206	5.13***
ventral mPFC	4	44	-4	854	5.38***
vmPFC	6	62	10		5.29***
vmPFC	-2	54	4		5.15***
**Nonsocial Prime > Target**					
R Superior Occipital Gyrus	18	-88	26	8410	7.70***
R Middle Occipital Gyrus	44	-74	16	1847	6.57***
L Middle Temporal Gyrus	-42	-64	8	282	4.92***
R Superior Temporal Gyrus	58	-6	-10	337	4.74***
L Middle Anterior Cingulate	-2	2	42	150	5.40**
R Middle Frontal Gyrus	48	46	8	286	5.26***
ventral mPFC	-2	56	4	1286	5.59***
vmPFC	8	50	0		5.36***
vmPFC	8	44	54		5.03***

Coordinates refer to the MNI (Montreal Neurological Institute) stereotaxic space. Whole-brain analysis thresholded at voxel-wise uncorrected *p* < 0.001 with cluster-wise FWE corrected *p* < 0.05, with voxel extent ≥ 10. Only the highest peaks of each cluster are shown. L = left, R = right. *p < 0.05, **p < 0.01, ***p < 0.001 (peak FWE corrected)

#### Retrieval Phase: Retrieving sequences of actions

Although we had no specific hypotheses on brain activity related to the retrieval of sequences, we conducted a number of whole-brain analyses for exploratory reasons.

We first examined differences between domains (i.e., social vs. nonsocial), but found no significant brain activations. We then contrasted differences between durations (20 s vs. 40 s) in all domains together and separately. The results demonstrated that the mPFC, left precuneus and calcarine gyrus were more strongly activated in the social 40-s compared with the social 20-s conditions, while no significant brain activation was found for the other contrasts. Moreover, exploring the interactions between Domain and Duration, we observed higher brain activity in the posterior cingulate cortex and precuneus in the cross-over interaction contrasts of (Social 40 s > Social 20 s) > (Nonsocial 40 s > Nonsocial 20 s) (Table 3).

**Table 3:** Whole-brain and parametric analysis of recognition of sequences during the retrieval phase

Contrasts and Anatomical Label	*MNI coordinate*			*Voxels*	*max t*
	*x*	*y*	*z*		
**Whole-brain analysis**					
**Social 20 s > Social 40 s**					
**---**					
**Social 40 s > Social 20 s**					
L Calcarine Gyrus	-14	-62	6	1837	5.38***
L Precuneus	-12	-42	44	243	4.15**
mPFC	8	34	18	254	5.20***
**Nonsocial 20 s > Nonsocial 40 s**					
---					
**Nonsocial 40 s > Nonsocial 20 s**					
---					
**Crossover Interaction: (Social 20 s < Social 40 s) > (Nonsocial 20 s < Nonsocial 40 s)**					
R Precuneus	10	−48	14	120	4.56*
L Posterior Cingulate Cortex	−10	−44	10	213	4.94**
**First-level parametric analysis with retrieval accuracy: positive correlation**					
**Social 20 s**					
R Lingual Gyrus	20	−66	−4	700	5.48***
R Precuneus	16	−66	24	476	4.78***
L Precuneus	−6	−64	38	389	4.78***
L ParaHippocampal Gyrus	−30	−34	−14	904	5.82***
R Postcentral Gyrus	26	−28	60	404	5.23***
R ParaHippocampal Gyrus	28	−26	−14	182	4.59**
L Thalamus	−10	−16	4	545	6.32***
L Posterior-Medial Frontal	−2	4	54	462	5.19***
L Superior Frontal Gyrus	−26	34	36	331	6.50***
**Social 40 s**					
R Middle Occipital Gyrus	34	−88	10	986	6.33***
L Middle Occipital Gyrus	−32	−76	20	578	5.08***
R Cerebellum (VI)	18	−70	-16	192	4.61**
R Superior Parietal Lobule	32	−44	58	159	5.45*
R Superior Temporal Gyrus	62	−34	10	156	4.78*
**Nonsocial 20 s**					
L Middle Occipital Gyrus	−16	−90	18	224	5.26**
R Middle Occipital Gyrus	30	−84	32	278	5.98***
L Middle Occipital Gyrus	−26	−74	24	172	5.22*
R Inferior Temporal Gyrus	46	−58	−8	310	5.68***
R Superior Parietal Lobule	20	−52	54	151	4.79*
**Nonsocial 40 s**					
L Putamen	−28	−8	4	149	5.72*
R Putamen	22	8	−6	146	5.06*
**Second-level regression analysis with retrieval accuracy**					
---					

Coordinates refer to the MNI (Montreal Neurological Institute) stereotaxic space. Whole-brain analysis thresholded at voxel-wise uncorrected *p* < 0.001 with cluster-wise FWE corrected *p* < 0.05, with voxel extent ≥ 10. Only the highest peaks of each cluster are shown. L = left, R = right

*p < 0.05, **p < 0.01, ***p < 0.001 (peak FWE corrected)

To investigate a potential relationship with retrieval accuracy, we conducted several parametric analyses. A first-level parametric analysis revealed that individuals’ retrieval accuracy was correlated with cerebellar lobule VI in the social 40-s condition (Table 3). No other effects of single conditions or contrasts were found. A second-level regression analysis showed no significant modulation either.

#### Metacognitive phase: Confidence on sequence retrieval

We conducted a whole-brain analysis and examined differences between domains (i.e., social vs. nonsocial) and between durations (20 s vs. 40 s) but found no significant brain activations.

**Fig. 3:**
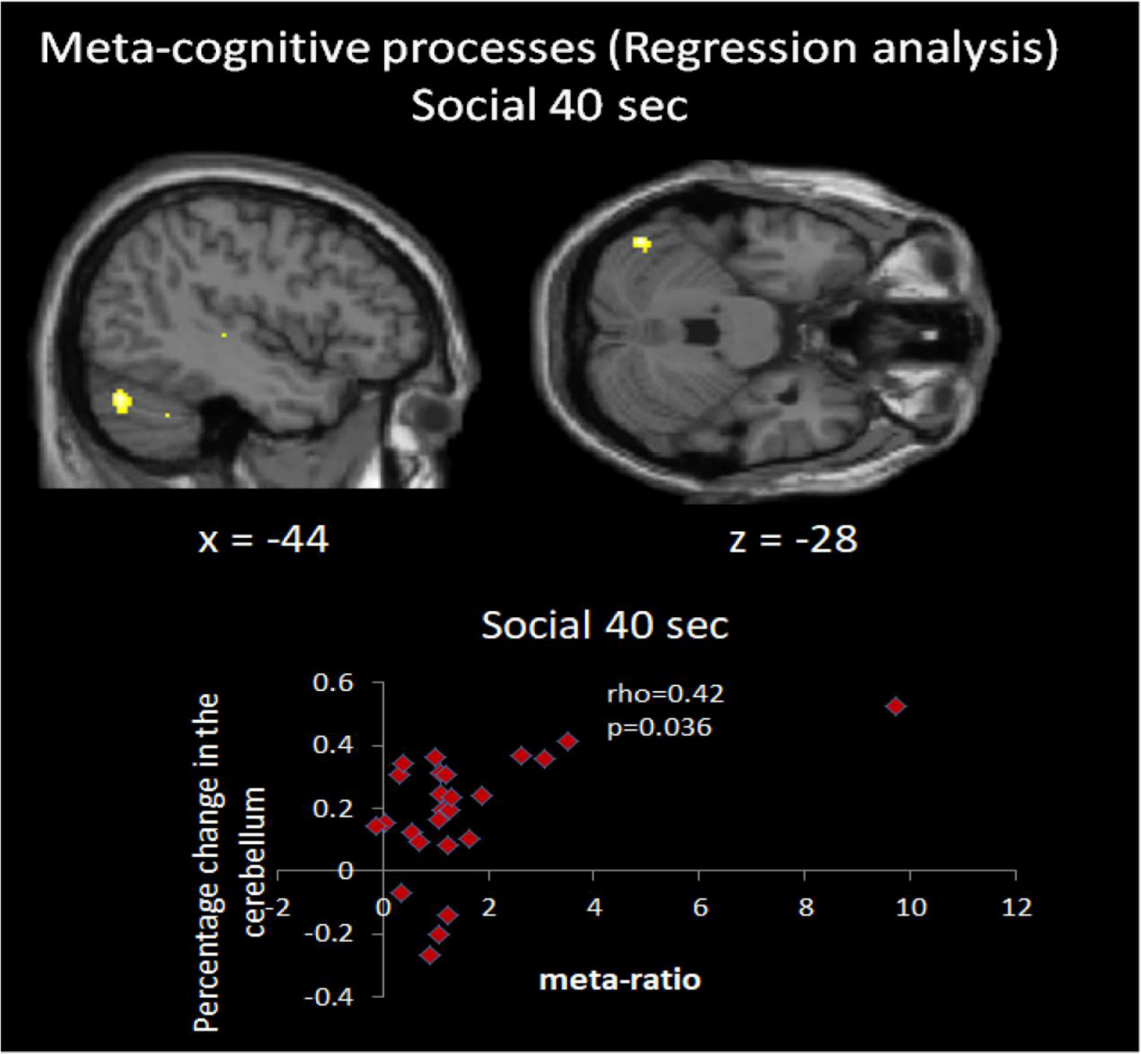
Second-level regression analysis: the cerebellar activation in social 40 sec condition shows covariation with individuals’ meta-cognitive sensitivity (i.e. meta-ratio; at an uncorrected threshold of *p* < 0.001). Top: Sagittal and Transverse views of brain activity in the left posterior cerebellum (Crus I), *p* < 0.05, FWE corrected. Bottom: Positive correlation between percentage signal change in the cerebellum and meta-ratio, *p* < 0.05

For the first-level parametric analysis, we found that confidence ratings in the social 20-s condition were positively correlated with cerebellar activation in Crus 2, lobule IX, and lobule VII and that confidence rating in the social 40-s condition were positively correlated with cerebellar activation in lobule IX and lobule VII. There also were correlations in the cortex (Table 4). A second-level regression analysis revealed that the individuals’ meta-ratio was correlated with cerebellar Crus 1 in the social 40-s condition (Table 4; Fig. 3). There also was a correlation with the superior parietal lobule during the nonsocial 20-s condition (Table 4). No other significant brain activations were found. Moreover, we found a significant positive correlation between the percentage signal change of cerebellar Crus 1 and the meta-cognitive index (i.e., meta-ratio), Spearman *rho* = 0.42, *p* = 0.036 (Fig. 3), which remained marginally significant after removing one outlier with an extremely high meta-ratio, Spearman *rho* = 0.35, *p* = 0.098. Note that we used Spearman instead of Pearson correlation because the percentage signal change variable was not normally distributed.

**Table 4.** Parametric analyses of retrieval confidence ratings

Contrasts and anatomical label	*MNI coordinate*			*Voxels*	*max t*
	*x*	*y*	*z*		
**First-level parametric analysis with Retrieval Confidence: positive correlation**					
**Social 20 s**					
R Cerebellum (Crus 2)	12	−76	−36	5,760	10.17***
R Cerebellum (VIII)	32	−68	−48		8.78***
R Cerebellum (IX)	14	−50	−44	301	6.38***
L Middle Occipital Gyrus	−16	−92	16	136	4.70*
R Precuneus	10	−58	32	131	5.41*
**Social 40 s**					
L Inferior Parietal Lobule	−38	−76	42	3,391	5.35***
L Superior Parietal Lobule	−26	−52	68	127	4.89*
L Precuneus	−10	−50	10	201	5.74**
R Cerebellum (IX)	8	−46	−40	316	5.72***
L Medial Cingulate Cortex (MCC)	−6	−38	36	205	4.74**
R ParaHippocampal Gyrus	38	−32	−14	128	4.54*
L Middle Temporal Gyrus	−66	−16	−12	653	6.15***
R Middle Temporal Gyrus	62	−6	−16	142	5.89*
L Caudate Nucleus	−6	14	2	486	6.03***
L Middle Frontal Gyrus	−22	26	46	163	5.29*
**Nonsocial 20 s**					
L Middle Occipital Gyrus	−26	−96	2	250	4.94**
R Lingual Gyrus	22	−82	-2	559	4.68***
**Nonsocial 40 s**					
R Posterior Cingulate Cortex (PCC)	12	−44	30	3929	7.12**
L Hippocampus	−34	−20	−14	216	7.14***
R Hippocampus	40	−16	−20	695	7.54***
L Middle Temporal Gyrus	−64	−12	−6	182	5.43**
R Middle Temporal Gyrus	62	−6	−12	202	6.15**
**Second-level regression analysis with meta ratio of retrieval confidence: positive correlation**					
**Social 20 s**					
**---**					
**Social 40 s**					
L Cerebellum (Crus 1)	−44	−70	−28	134	5.13*
**Nonsocial 20 s**					
L Superior Parietal Lobule	−18	−44	72	303	6.61***
**Nonsocial 40 s**					
**---**					

Coordinates refer to the MNI (Montreal Neurological Institute) stereotaxic space. Whole-brain analysis thresholded at voxel-wise uncorrected *p* < 0.001 with cluster-wise FWE corrected *p* < 0.05, with voxel extent ≥10. Only the highest peaks of each cluster are shown. L = left, R = right

*p < 0.05, **p < 0.01, ***p < 0.001 (peak FWE corrected)

#### Metacognitive phase: Confidence on trait attributions

None of the differences between Domain (i.e., social vs. nonsocial) or Duration (20 s vs. 40 s) were significant, although the superior frontal gyrus, cerebellum (Crus I), middle occipital gyrus, and postcentral gyrus were activated in the nonsocial 40 > 20-s contrast.

## Discussion

Trait attribution is important for human social interaction. In our daily interactions, our impression of others is not dependent on a single event, and people dynamically integrate a variety of information, such as others’ behaviors to make social inferences. In this, the sequence of behaviors might be very important. In the current study, we investigated whether the posterior cerebellum is involved in learning specific sequences of actions describing a trait of another person, although these actions do not necessarily form a logical succession. Our findings revealed, for the first time, significant activation in the posterior cerebellum (Crus 2) while learning the sequences of actions relevant for social trait inference, and that this activation was stronger than for nonsocial objects sequences. Moreover, we also found that metacognitive sensitivity in recollecting these action sequences engaged the posterior cerebellum (right Crus 1).

### Posterior cerebellum and learning social action sequences

At the study phase, consistent with our hypothesis, we found a general effect of significant cerebellar activation in the Crus 2 when participants were learning a given sequence compared with a nonsequential control which involved simply reading the nonsocial sentences. Although this effect was found in both social and nonsocial domains, directly comparing sequence learning of social versus nonsocial sentences confirmed that the posterior cerebellar Crus 2 is predominantly recruited during social action sequences. This confirms the functional role of the cerebellum in social sequence detection and learning. Note that it is quite unlikely that the stronger activation of social sequence learning is simply due to higher memory load and executive effort required for social sentences in comparison with nonsocial sentences, because retrieval accuracy was almost identical across these two conditions, and activation was opposite to that reported for higher verbal memory load in recent research (deactivation: Peterburs et al., 2019).

Other potential differences between these conditions, such as a greater motivational and emotional impact of social sentences might contribute to the stronger activation of the posterior cerebellum, although these are inherent (and often desired) aspects of social action, which cannot easily be eliminated. Controlling such effects would not only be difficult but would probably also eliminate the very social nature of these actions. Attempts have been made in the past to find an underlying common theoretical ground for social and nonsocial neural processes, but they were largely discredited (Van Overwalle, 2011). However, although stronger for social material, activation was revealed in the same cerebellar area for both social and nonsocial domains, presumably because of the common mental sequencing process in both conditions. Taken together, we cannot exclude the possibility that the present social distinction might perhaps rather be graded than categorical, and might involve other aspects besides the social nature of the material. This is a question for future research.

One may argue that it might be have been helpful to introduce other control tests, such as a social nonsequencing task in addition to the current nonsocial nonsequencing task, so that the social and nonsocial sequencing conditions could have been compared directly with their nonsequencing control counterparts. In this way, the above concerns would have played less a role, and stronger cerebellar activation for social sequencing compared with social nonsequencing would have strengthened our hypothesis that the posterior cerebellum is predominantly recruited in social processing. This is certainly correct. However, given the time-constraints imposed by having participants having to lie still in the scanner, we opted for less control conditions. And yet, when considering the three main conditions (social sequencing, nonsocial sequencing, and nonsocial nonsequencing conditions), all comparisons of the social sequencing condition with appropriate control conditions are possible, if not directly, then at least indirectly. First, to test whether the sequencing role of cerebellum is specific to social mentalizing, a nonsocial sequencing control condition was included. Second, to test whether sequencing was critical for cerebellar activation, we included the nonsocial nonsequencing control condition. Another reason why we did not include a social nonsequencing condition is that trait-implying behavioral sentences might potentially activate the cerebellum to some degree even without explicit sequencing (because the implied actions might activate the cerebellum somewhat), and therefore, this condition would not constitute an entirely valid control (Van Overwalle et al., 2014).

Our findings are consistent with recent research investigating the role of the cerebellum in action sequences during mentalizing. In social cognition, Van Overwalle et al. (2019a, b, 2020a) found that cerebellar patients were strongly impaired in generating the correct sequence of social actions that required the understanding of other’s beliefs compared with healthy control participants. A recent fMRI study in which healthy participants were instructed to generate the correct chronological order of cartoon-like stories, confirmed that the posterior cerebellum (Crus 2) was involved in generating new action sequences requiring the understanding of others’ beliefs compared to routine social and nonsocial events (Heleven et al., 2019).

Our study extended these studies by exploring the functional role of the posterior cerebellum in sequencing processing during social trait judgment. Traits require high-level mentalizing that reflects a person’s permanent internal state abstracted from a range of behavioral descriptions. However, as earlier studies demonstrate, trait inferences alone seem not to recruit the posterior cerebellum systematically. Only when the sequential order of actions implying trait inferences is highlighted, as in the present study, then the posterior cerebellum is robustly engaged. This is in line with previous studies showing that social mentalizing tasks without a clear sequencing component do not robustly elicit the cerebellar activation (Hoche, Guell, Sherman, Vangel, & Schmahmann, 2016; Sokolovsky, Cook, Hunt, Giunti, & Cipolotti, 2010).

Moreover, social actions and language are inherent to social interaction. Research found posterior cerebellar activation also in language sequencing tasks. For example, completion of predictive sentences increased activation in the right posterior cerebellum (D’Mello, Turkeltaub, & Stoodley, 2017; Lesage, Morgan, Olson, Meyer, & Miall, 2012). It is currently unclear how exactly to interpret this common posterior cerebellar activation, because some language studies involve social stories with human actors, just as in the present experiment. Moreover, a recent meta-analysis indicated that the posterior Crus 2 is activated predominantly under social manipulations, and much less so under linguistic manipulations (Van Overwalle, Ma, & Heleven, 2020b). Separating social from purely linguistic processes is a pressing issue for future research.

A novel contribution of our study is that the sequences involved a larger time window (i.e., several separate events that implied the same trait) and did not form a logical order as when they are an inherent part of one single event as in earlier research (Heleven et al., 2019; Van Overwalle, De Coninck, et al., 2019a). As such, this study is more representative of the complexities or modern social life, where we receive many pieces of information on people’s behavior via small talk, gossip, and social media. As social interaction continues, dynamic updating of the inferences based on new information may be required (Mende-Siedlecki, Cai, & Todorov, 2013).

A potential limitation in the interpretation of our results is that social sentences and object descriptions differed in complexity and concreteness and that such differences may have confounded the effects. However, this explanation is unlikely. The behavioral data do not indicate differences in complexity, because accuracy was around 75%. Moreover, this number reveals imperfect performance, so that lack of behavioral differences is not due to a ceiling effect. Another restriction of the present study was that all actions implied the same trait, so that the sequence of the actions was unrelated to the trait. However, what would happen if this was not the case, and actions would imply distinct inconsistent traits presented in various orders? This would definitely trigger distinct trait inferences (e.g., impulsiveness when hurting somebody first versus lawfulness when hurting in an act of self-defense). The interaction between action sequences and distinct trait inferences, and the role of the posterior cerebellum, is a promising direction for future research.

Apart from cerebellar areas, during social sequence learning we found stronger activations in several cortical areas including the mPFC, TPJ, precuneus in comparison with nonsocial sequence learning. These cortical areas are critical during social mentalizing (see meta-analysis by Schurz, Radua, Aichhorn, Richlan, & Perner, 2014; Van Overwalle & Baetens, 2009). These findings provide evidence that social mentalizing was going on during the processing of the trait-implying sentences. We also found stronger activation in the hippocampus and medial temporal lobe, which traditionally have been associated with the encoding of declarative memory (Gabrieli, Brewer, Desmond, & Glover, 1997), and its interaction with the prefrontal cortex is crucial for successful encoding of novel information (Simons & Spiers, 2014).

When taking into account the duration of the study phase, we found stronger posterior cerebellum (Crus 2) activation when participants had more time (40 s instead of 20 s) to learn the order of the action sequences in the social domain. Note that the time window to statistically analyze these two conditions was identical under all experimental conditions (i.e., initial 10 s). This implies that not the duration itself, but rather the expected total duration, may have engaged a different learning process. One possible explanation is that a longer anticipated duration may contribute to a better integration and thus learning of sequences in the initial stages of learning. Another related explanation is that it may have led to deeper and more engaged learning from the start. Note, however, that meta-cognitive confidence itself did not differ between conditions, although this lack of difference should be treated with caution, since our standard deviation was quite large.

### Repetition suppression and trait representations

The suppression of activation in the mPFC for person traits (and by extension here also for object features) is in line with earlier studies on trait repetition suppression (Heleven, Boukhlal, & Van Overwalle, 2018; Ma et al., 2014) and therefore indicates that trait inferences were being made while learning the sequences of actions. The mPFC is thought to be the key brain area involved in the trait judgments based on behavioral descriptions (see meta-analyses by Schurz et al., 2014; Van Overwalle & Baetens, 2009). As revealed in earlier neuroimaging studies using trait repetition suppression, the neural code of traits is represented in the ventral part of mPFC (Heleven & Van Overwalle, 2018; Ma et al., 2014). Our finding of trait suppression in the vmPFC strongly supports the important role of the vmPFC in representing and inferring traits of other persons. The fact that we also found repetition suppression in the vmPFC for nonsocial judgments may be due to the present stimulus material, which involved object features that required high-level abstractions from the behavioral material. Previous research revealed that the mPFC is activated for inferences, which require high-level abstractions and categorical judgments, across social as well as for nonsocial objects, as long as the construal level is high (Baetens, Ma, Steen, & Van Overwalle, 2014; Baetens, Ma, & Van Overwalle, 2017).

### Frontal and parietal brain regions and retrieving social sequences

Recall that we had no particular hypotheses concerning sequence retrieval, because cerebellar sequence theories suggest that the main function of the cerebellum is to reduce prediction errors (Caligiore et al., 2019), and hence the cerebellum recruits the highest activation while learning novel sequences, and much less during retrieval of already learned sequences. Consistent with this, we found no differences in cerebellar activation during retrieval of social versus nonsocial events. A parametric analysis revealed a relationship of individuals’ retrieval accuracy in the social 40-s condition and activation in the posterior cerebellar lobule VI (MNI coordinates 18 -70 -16), which is part of ventral attention network (Buckner et al., 2011). There were no other effects in the cerebellum. Together, these results show that retrieval did not recruit the mentalizing cerebellum.

However, at the cerebral cortex, we found that the mPFC was recruited more during social sequences of 40 s than 20 s. This fits with the role of the area in the trait inference process. Interestingly, the precuneus also was most strongly recruited in this contrast (Table 3). The precuneus is thought to be involved in successful episodic memory retrieval (Dörfel, Werner, Schaefer, Von Kummer, & Karl, 2009; Lundstrom et al., 2003). Given that this cortical area also is involved in social mentalizing (Schurz et al., 2014; Van Overwalle & Baetens, 2009), the episodic memory retrieval processes in this experiment were arguably more strongly recruited while recollecting social event sequences at a deeper level (40 > 20 s).

### Metacognitive confidence on retrieving sequences

We hypothesized that metacognitive confidence on one’s memory of the sequences would recruit frontal cortical areas as revealed in prior research (Vaccaro & Fleming, 2018), potentially with the aid of local activations in posterior cerebellar areas involved in sequencing. Note that this hypothesis is not necessarily in contradiction with our lack of hypothesized activation during retrieval itself, because confidence in accuracy requires to assess implicitly or explicitly the amount of errors during retrieval, which is consistent with the main function of error reduction as hypothesized by sequence theories.

Partly in line with these predictions, metacognitive confidence at the sequence retrieval task revealed a significant correlation between activation in Crus 2 in the social 20-s condition at trial level, and between Crus 1 and participants’ meta-cognitive index (i.e., meta-ratio) in the social 40-s condition, and a marginally significant positive correlation between the percentage signal change of cerebellar Crus 1 and the meta-ratio in this condition.

Our results did not confirm the involvement of frontal cortical areas, although a recent meta-analysis of 36 metacognition studies indicated that a domain-general network, including the medial and lateral prefrontal cortex, was associated with level of confidence in self-performance (Vaccaro & Fleming, 2018). However, the results are in line with our suggestion that task-specific processes related to sequencing might be recruited when making meta-cognitive judgments on this process. Of note, we found that metacognitive sensitivity was correlated with individual differences in alexithymia, a measure of metacognitive awareness of self-experienced emotionality. Specifically, alexithymia refers to difficulties in describing the feeling of oneself (Sifneos, 1973). More recently, alexithymia has been linked to problems in mentalization and metacognition (Babaei, Gharechahi, Hatami, & Varandi, 2015; Lysaker et al., 2014). Our results may indicate that individuals with difficulties in consciously identifying their feelings also have related metacognitive difficulties in identifying how well they performed on the retrieval of action sequences.

## Conclusions

The present findings highlight for the first time the important role of the posterior cerebellar Crus 2 in learning and encoding sequences of trait-implying human actions without an inherently logical succession. This confirms the hypothesis recently put forward by Leggio et al. (2015) that the posterior cerebellum is strongly involved in identifying and learning sequences of social actions and extends this for actions that imply the trait of others in a larger social context.
